# High-Throughput Multi-Analyte Luminex Profiling Implicates Eotaxin-1 in Ulcerative Colitis

**DOI:** 10.1371/journal.pone.0082300

**Published:** 2013-12-18

**Authors:** Lori A. Coburn, Sara N. Horst, Rupesh Chaturvedi, Caroline T. Brown, Margaret M. Allaman, Brooks P. Scull, Kshipra Singh, M. Blanca Piazuelo, Maithili V. Chitnavis, Mallary E. Hodges, Michael J. Rosen, Christopher S. Williams, James C. Slaughter, Dawn B. Beaulieu, David A. Schwartz, Keith T. Wilson

**Affiliations:** 1 Department of Medicine, Division of Gastroenterology, Hepatology, and Nutrition, Vanderbilt University Medical Center, Nashville, Tennessee, United States of America; 2 Department of Pathology, Microbiology, and Immunology, Vanderbilt University Medical Center, Nashville, Tennessee, United States of America; 3 Department of Cancer Biology, Vanderbilt University Medical Center, Nashville, Tennessee, United States of America; 4 Veterans Affairs Tennessee Valley Healthcare System, Vanderbilt University Medical Center, Nashville, Tennessee, United States of America; 5 Department of Pediatrics, Gastroenterology, Hepatology, and Nutrition, Vanderbilt University Medical Center, Nashville, Tennessee, United States of America; 6 Department of Biostatistics, Vanderbilt University Medical Center, Nashville, Tennessee, United States of America; Charité, Campus Benjamin Franklin, Germany

## Abstract

Accurate and high-throughput technologies are needed for identification of new therapeutic targets and for optimizing therapy in inflammatory bowel disease. Our aim was to assess multi-analyte protein-based assays of cytokines/chemokines using Luminex technology. We have reported that Luminex-based profiling was useful in assessing response to L-arginine therapy in the mouse model of dextran sulfate sodium colitis. Therefore, we studied prospectively collected samples from ulcerative colitis (UC) patients and control subjects. Serum, colon biopsies, and clinical information were obtained from subjects undergoing colonoscopy for evaluation of UC or for non-UC indications. In total, 38 normal controls and 137 UC cases completed the study. Histologic disease severity and the Mayo Disease Activity Index (DAI) were assessed. Serum and colonic tissue cytokine/chemokine profiles were measured by Luminex-based multiplex testing of 42 analytes. Only eotaxin-1 and G-CSF were increased in serum of patients with histologically active UC vs. controls. While 13 cytokines/chemokines were increased in active UC vs. controls in tissues, only eotaxin-1 was increased in all levels of active disease in both serum and tissue. In tissues, eotaxin-1 correlated with the DAI and with eosinophil counts. Increased eotaxin-1 levels were confirmed by real-time PCR. Tissue eotaxin-1 levels were also increased in experimental murine colitis induced by dextran sulfate sodium, oxazolone, or *Citrobacter rodentium*, but not in murine *Helicobacter pylori* infection. Our data implicate eotaxin-1 as an etiologic factor and therapeutic target in UC, and indicate that Luminex-based assays may be useful to assess IBD pathogenesis and to select patients for anti-cytokine/chemokine therapies.

## Introduction

The specific etiology of inflammatory bowel disease (IBD) has yet to be identified. The chronic relapsing and remitting mucosal inflammation associated with the two main variations, ulcerative colitis (UC) and Crohn’s disease, affects approximately 1.4 million Americans leading to significant morbidity and decreased quality of life [1], [2]. There is hope that from ongoing investigations of the mechanisms underlying the abnormal immune system response in these patients, markers of disease severity or even new therapeutics will be identified. One area of focus is the assessment of various inflammatory mediators, including proinflammatory cytokines, which have been implicated in the pathophysiology of IBD [3], [4].

Platforms such as genome-wide association studies (GWAS) have identified multiple immunoregulatory genes associated with IBD susceptibility [5]. This includes genes related to cytokine/chemokine signaling, which were recently further evaluated retrospectively in colonic tissues from IBD patients and control subjects via assessment by polymerase chain reaction (PCR) to delineate a potential signaling pathway profile [6]. In the current study, we hypothesized that multiplex Luminex technology could be used to identify differences in serum and tissue cytokines and/or chemokines from a prospective cohort of UC patients and control subjects. Bead-based Luminex assays allow for the analysis of multiple protein analytes in a single aliquot of serum or tissue lysate. This may allow for the identification of a larger profile panel with which to assess IBD patients, may identify specific targets for future therapeutics, or allow for more sensitive monitoring of patient responses to therapy. We have recently demonstrated the utility of Luminex-based assays in the analysis of cytokine/chemokine profiles in mouse models of colitis [7], [8], [9], [10], including the effects of altered L-arginine availability [10] and benefits of L-arginine supplementation in dextran sulfate sodium-induced colitis [8].

We now report that in a prospective study of 42 analytes tested by Luminex profiling, 13 cytokines/chemokines were increased in tissues with active UC vs. non-UC controls, but only 2, eotaxin-1 and G-CSF, were increased in the serum of patients with active UC. Of these 2 markers, only eotaxin-1 was increased at all levels of active disease in both serum and UC tissues. Tissue eotaxin-1 levels correlated with disease activity and with eosinophilic infiltration into tissues. Also known as CCL11, eotaxin-1 is a chemoattractant for eosinophils and is expressed in multiple tissues in humans including the skin, the respiratory epithelium and the intestine [11]. Our Luminex profiling implicates eotaxin-1 in the pathophysiology of UC and provides rationale for new therapeutic strategies targeting this chemokine.

## Materials and Methods

### Human subjects

The study protocol was approved by the Institutional Review Board at Vanderbilt University. Written informed consent was obtained from all subjects for analyses of demographics and medical history as well as for serum and tissue biopsies obtained at the time of endoscopic procedures as a part of the clinical trial “Effects of L-Arginine in Colitis and Colon Cancer”, identifier NCT01091558 (clinicaltrials.gov).

Subjects were prospectively recruited in the clinic or endoscopy unit at Vanderbilt University Medical Center prior to outpatient colonoscopy for either colorectal cancer screening or UC surveillance purposes between September 2009 and September 2011. Patient participation in the current study ended after the colonoscopy. The enrollment target of 200 subjects with a goal of a minimum of 30 control subjects and 140 UC patients that would complete the study was based on the predicted number of eligible patients that could be enrolled during the recruitment period. Exclusion criteria for the study were: pregnancy, known coagulopathy or bleeding disorders, known renal or hepatic impairment, history of organ transplantation, or unable to give informed consent. All subjects underwent overnight fast and received polyethylene glycol electrolyte solution for bowel preparation prior to colonoscopy. In total, we enrolled 204 subjects of which 29 were excluded from the final analysis for the following reasons: 7 subjects had pathology more consistent with Crohn’s disease, 12 subjects were deemed ineligible for the study protocol biopsies at the time of colonoscopy by the endoscopist either due to poor bowel preparation or the finding of very severe disease that was felt to increase the risk of complications related to the study biopsy protocol, 3 subjects were screened incorrectly and met the exclusion criteria above, 2 subjects withdrew consent, 1 subject underwent colectomy prior to undergoing the study colonoscopy, 2 patients were consented but did not undergo colonoscopy during the study period, 1 control patient was excluded due to treatment with prednisone for a possible rheumatologic disorder, and 1 patient was excluded from the control group for use of hydrocortisone suppositories for possible UC that on further assessment was determined to have irritable bowel syndrome. The remaining 175 subjects that completed the study protocol were 38 control subjects and 137 UC patients.

All of these patients consented to undergo serum collection as well as three additional tissue biopsies for research purposes in four colonic segments (ascending, transverse, descending and rectum) at the time of scheduled colonoscopy. Study serum and tissue biopsies were snap frozen with dry ice and then stored at −80°C. Surveillance biopsies from UC patients were reviewed by the Department of Pathology at Vanderbilt University and graded accordingly as: normal, quiescent, mild, moderate, or severe activity. The Mayo Disease Activity Index (DAI) was determined for UC patients at the time of colonoscopy by standard measures (0 – 12 scale), which included the number of bowel movements per day, the presence or absence of blood, endoscopic severity score, and physician’s global assessment [12]. Endoscopic severity was determined by gastroenterologists specializing in IBD (D.A.S.; D.B.B.; S.N.H.) using the following scale: normal, mild disease (erythema, decreased vascular pattern, mild friability), moderate disease (marked erythema, lack of vascular pattern, friability, erosions), or severe disease (spontaneous bleeding, ulceration). All associated study data were collected and managed using Research Electronic Data Capture (REDCap) electronic data capture tools hosted at Vanderbilt [13].

### Assessment of human serum and tissue cytokines/chemokines

Human serum and tissue samples were obtained as above and then assessed using Milliplex™ MAP (Millipore, Billerica, MA) multiplex magnetic bead-based antibody detection kits according to the manufacturer’s protocols. Tissues were lysed in radioimmunoprecipitation assay (RIPA) buffer with a mortar and pestle-type rotary homogenizer, and used for Luminex assay on a FLEXMAP 3D**®** machine as described [7], [8], [9], [10], [14]. Protein concentration was measured in the tissue lysates using the bicinchoninic acid (BCA) method [8], [15].

We performed our initial analyses of the serum and tissue samples with a 42 analyte (42-plex) kit. However, there were 3 separate sets of beads for the analytes RANTES, PDGF-AA, and PDGF-AB/AA that were not pre-mixed and these caused significant clumping during the assays, so we eliminated these 3 analytes, leaving 39 analytes. After assaying approximately half of the samples (in serum, *n* = 16 for controls and 57 for UC; in tissue, *n* = 20 for controls and 42 for UC), we found that 10 of the analytes consistently showed no difference between UC and control subjects. Therefore, for the remaining samples (an additional *n* = 18 for controls and 52 for UC in serum; *n* = 14 for controls and 44 for UC in tissue), 29 analytes were assessed. Details of the number of cases assessed for each of the analytes are provided in the legends to the data tables and figures. In general, there were fewer UC tissue samples assessed by Luminex assay than serum samples, as some of the tissue samples were used for other assessments such as mRNA expression. In addition, we also utilized a custom Milliplex™ kit to measure levels of eotaxin-2, eotaxin-3, and IL-23, resulting in a final total of 42 protein targets that were analyzed.

### mRNA analysis

mRNA was isolated as described [8], [16]. Then, 1 µg of RNA was reverse-transcribed using an iScript cDNA synthesis kit (Bio-Rad, Hercules, CA, USA). Each PCR reaction was performed with 1 µl of cDNA and 2x LightCycler® 480 SYBR Green I Master Mix (Roche, Indianapolis, IN, USA). Primers for β-actin were used as described [17]. The sequences for human eotaxin-1 primers were as follows: (F) 5' TGAAGCTTGGGCCTTCTGTCCCAACC 3', and (R) 5' GGTCGACTGGAGTGAGATTTTTGGTC 3'. The sequences for mouse eotaxin-1 primers were as follows: (F) 5' TGCAGAGCTCCACAGCGCTTC 3', and (R) 5' TGAGCCAGCACCTGGGAGGT 3'. The thermal cycling conditions and the methods used to calculate relative expression have been described previously [18], [19], [20].

### Tissue eosinophil assessment

Hematoxylin and eosin (H&E) stained slides for each of our subjects were obtained from the Vanderbilt Tissue Pathology Shared Resource. The slides were examined in a blinded manner by a gastrointestinal pathologist (M.B.P.). Tissues were scored as the number of eosinophils in the colonic mucosa per high-power field (HPF) using a Nikon Eclipse E200 microscope with a 40x objective. A total of 5 HPFs were assessed for each biopsy, with each subject having between 1 and 6 biopsies. Areas with lymphoid follicles were avoided. The scores were then averaged for each subject.

### Animals

All animal studies were carried out according to an approved Institutional Animal Care and Use Committee protocol at Vanderbilt University. Six-week-old wild-type (WT) C57BL/6 mice were purchased from The Jackson Laboratory (Bar Harbor, ME). Only male mice were used for experiments, and all mice were used at 7 weeks of age and maintained under biosafety level 2 conditions.

### Induction of DSS colitis in mice

Dextran sulfate sodium (DSS, MW 36,000−50,000, MP Biomedical, Solon, OH) was added to the drinking water of the mice for 7 days as described [8], [16]. The final concentration of DSS was 4% (wt/vol). Mice were allowed free access to water during the experiment. At day 7, the animals were sacrificed and the colons were removed, cleaned, and two proximal and two distal 2 mm^2^ samples were reserved, such that one from each region was combined and snap-frozen.

### Induction of oxazolone colitis

Oxazolone colitis was induced as previously described [9]. Briefly, anesthetized mice were sensitized by topically applying 3% oxazolone (4-ethoxymethylene-2-phenyl-2-oxazolin-5-one; Sigma-Aldrich, Saint Louis, MO) in 100% ethanol (150 µl) on the shaved abdomen. Seven days later, 2% oxazolone in 50% ethanol (150 µl) was administered intrarectally with a 5 French plastic infant feeding tube (C. R. Bard, Covington, GA). Control mice were treated with ethanol vehicle alone. Mice were sacrificed 3 days after rectal oxazolone administration, the colons were removed, cleaned, and samples were snap-frozen as described above.

### Induction of *Citrobacter rodentium* colitis

Mice were orally inoculated with wild-type *C. rodentium* as described previously [16], [21], [22]. Briefly, bacteria were grown overnight in Luria broth and mice were infected by oral gavage with 0.1 ml of broth containing 1×10^8^ colony-forming units of *C. rodentium*. Control mice received sterile broth. At day 14, the animals were sacrificed and the colons were removed, cleaned, and samples were snap-frozen as described above.

### Induction of *Helicobacter pylori* gastritis

*H. pylori* SS1, a mouse-adapted human strain [23] was grown on blood agar plates under microaerobic conditions as described [24]. Concentrations of bacteria were determined by optical density at 600 nm. Mice were inoculated with 5×10^8^ colony-forming units of *H. pylori* SS1 in 0.1 mL of Brucella broth, or broth vehicle control, every other day 3 times by oral gavage, and gastric tissues were harvested 4 months later [15], [24].

### Statistical Analysis

Quantitative data are shown as the mean ± SD. Outlier testing using the Grubbs method, also called the extreme studentized deviate method, and statistical analyses were performed with GraphPad Prism 5.0 (San Diego, CA). For human data, where two groups were compared, unpaired data was evaluated by Mann-Whitney U test, while paired data was evaluated by Wilcoxon signed rank test. Data with more than two groups were first analyzed by Kruskal-Wallis H test (nonparametric ANOVA) and if *p*<0.01, then pairwise comparisons using the Mann-Whitney U test were performed, where *p*<0.05 was considered statistically significant. For categorical data we used Pearson's χ^2^ test when no more than one expected cell count was less than 5. Correlations were determined by Spearman's rank correlation coefficient. For animal data, pairwise comparisons using the Mann-Whitney U test were performed.

## Results

### Patient characteristics

To test the hypothesis that cytokine and chemokine profiles can be used to assess UC patients, we analyzed serum and colonic tissue expression of a panel of cytokines and chemokines in human subjects. Prospectively collected samples from control subjects and patients with UC of varying histologic disease severity were used. The demographic information for the 38 control subjects and 137 UC patients used in this study is shown in Table 1. Notably, there were no significant differences in gender distribution or smoking between groups. However, the UC patients were significantly younger than the control subjects, as the majority of the control subjects were being evaluated for colon cancer screening or other non-IBD related indications. On histologic evaluation of the 137 UC patients, 41 had quiescent disease, 44 had mild disease, 31 had moderate disease, and 21 had severe disease. Of the 41 UC patients with quiescent disease, there were 17 with normal histopathology in all biopsies from the study colonoscopy. The body mass index was significantly lower in patients with active UC than in control subjects. More than 90% of the UC patients were on therapy for IBD. The majority of patients were on 5-aminosalicylate therapy. There was more corticosteroid use in the active UC group when compared to the histologically quiescent group. Interestingly, there was more anti-TNF-α use in the histologically quiescent group compared to the active UC group (Table 1).

**Table 1:** Demographic features of the study subjects.

	Control (*n* = 38)	Quiescent UC (*n* = 41)	Active UC (*n* = 96)
Age, mean ± SD	51.1±12.8	42.2±13.3^b^	41.7±14.2^c^
Gender	39.5% Male	39.0% Male	41.7% Male
Body Mass Index, mean ± SD	28.6±4.8	28.0±6.4	26.7±5.9^b^
Smoking (% of total)	5 (15.2%)	1 (2.5%)	5 (5.2%)
Any IBD Therapy (% of total)	1 (2.6%)	40 (97.6%)^c^	89 (92.7%)^c^
5-aminosalicylates (% of total)	1 (2.6%)*	33 (80.5%)^c^	75 (78.1%)^c^
Corticosteroids (% of total)	0	5 (12.2%)	32 (33.3%)^c,d^
Immunomodulators (% of total)	0	16 (39.0%)^c^	28 (29.2%)^c^
Anti-TNF-α (% of total)	0	14 (34.1%)^c^	14 (14.6%)^a,d^

^a^ *p*<0.05, ^b^ *p*<0.01, ^c^ *p*<0.001 vs. control; ^d^ *p*<0.05 vs. quiescent UC. *This patient was started on therapy for presumed UC but with normal endoscopic evaluation was felt to have irritable bowel syndrome. Age and body mass index statistics were assessed by the Kruskal-Wallis test followed by the Mann-Whitney test. Categorical data were analyzed using the Pearson’s χ^2^ test. UC status determined by histologic activity.

### Eotaxin-1 and G-CSF are the only cytokines/chemokines increased in the serum of patients with active UC vs. control subjects

**Figure 1:**
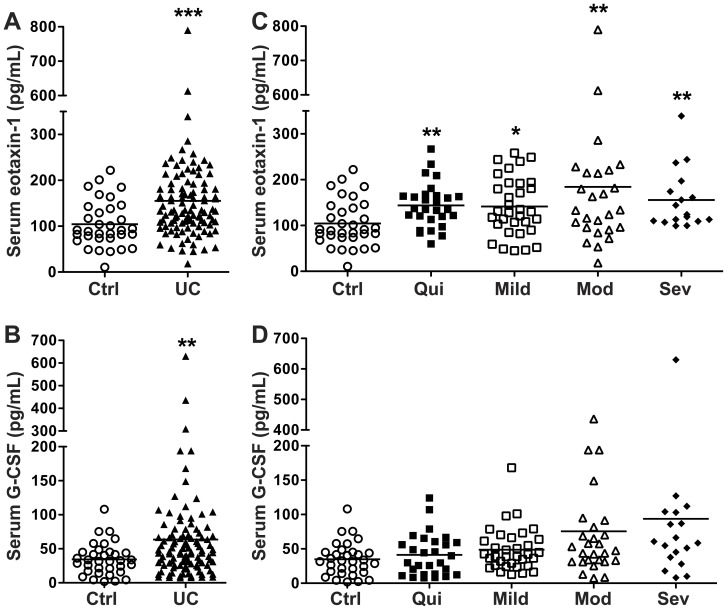
Serum analytes increased in UC, as measured using Luminex technology. Blood was obtained from each subject and processed within 30 min to obtain serum. Disease categories are based on maximal tissue histopathology. *n* = 34 for control (Ctrl) and *n* = 109 for UC. (A) Eotaxin-1 and (B) G-CSF concentrations in control and all UC patients. (C) Eotaxin-1 and (D) G-CSF concentrations in sera from UC patients stratified by histologic disease severity, and from control subjects. **p<0.01; ***p<0.001 vs. control.

Multi-analyte testing using Luminex technology to profile serum cytokines/chemokines revealed that 2 analytes out of 42 tested were increased in patients with active UC vs. control subjects: eotaxin-1 and G-CSF (Table 2). These increases compared to non-UC controls persisted when all patients with either active or quiescent UC were included (Figure 1A and 1B). When UC patients were grouped based on their maximal tissue histopathology, we found that eotaxin-1 was increased at all levels of disease activity (Figure 1C). In contrast, G-CSF was not significantly increased at any specific level of histologic activity (Figure 1D).

**Table 2:** Serum cytokine/chemokine profiles.

	Control	Quiescent UC	Active UC
Eotaxin-1^a^	104.4±50.3	143.9±48.4**	159.2±110.0***
G-CSF^a^	34.5±23.5	41.4±30.5	71.4±90.4**§
GM-CSF^a^	14.0±17.6	21.7±17.5	23.1±29.2
IP-10^a^	355.3±146.5	388.3±260.5	392.1±210.6
TNF-α^a^	9.8±5.6	8.4±4.3	11.5±6.9
TNF-β^a^	3.3±3.5	5.2±7.5	4.8±4.3
MCP-1^a^	442.0±183.6	476.2±215.8	422.0±183.4
MIP-1α^a^	13.4±14.5	6.6±5.1	18.9±18.9
MIP-1β^a^	40.1±37.6	30.7±17.7	44.7±51.7
IL-1α^a^	51.3±70.9	16.3±15.1	45.1±78.8
IL-1β^a^	4.3±3.7	7.1±8.3	7.2±8.9
IL-1RA^a^	26.5±20.3	36.2±59.4	52.6±52.2
IL-2^a^	3.8±2.2	6.7±5.1	9.9±17.9
IL-3^a^	0.1±0.0	1.5±0.0	0.6±0.4
IL-4^a^	17.0±16.6	8.1±6.3	17.6±32.7
IL-5^a^	1.5±1.8	1.8±2.4	1.0±1.5
IL-6^a^	9.7±10.7	4.5±3.6	9.6±9.7
IL-7^a^	8.0±9.8	3.8±2.3	10.4±19.6
IL-8^a^	36.6±51.9	14.4±7.6	38.7±42.4*§§§
IL-9^a^	1.7±1.3	2.3±2.0	4.9±3.4
IL-10^a^	6.8±6.7	22.4±25.4	21.0±23.4
IL-12p40^a^	26.6±30.0	26.0±26.3	44.1±46.4
IL-12p70^a^	5.6±7.9	3.6±2.3	12.0±26.5
IL-13^a^	10.4±15.9	3.5±4.6	10.7±17.1
IL-15^a^	3.0±2.4	5.4±3.4	6.2±5.4
IL-17^a^	65.1±98.4	5.2±6.5*	56.3±131.3§§
VEGF^a^	313.9±504.8	109.7±91.0	284.1±386.7
EGF^a^	86.8±65.0	52.5±32.5	68.6±44.9
IFN-γ^a^	80.4±153.4	12.1±15.5*	81.1±162.8§§§
IFN-α2^a^	20.6±21.6	15.4±10.5	18.6±24.0
FGF-2^b^	59.9±61.3	33.9±26.4	71.0±85.4
TGF-α^b^	1.8±1.1	2.5±1.7	11.3±32.4
FLT-3L^b^	10.2±9.6	31.8±27.4	17.5±12.5
Fractalkine^b^	59.7±57.4	32.0±45.3	301.6±518.7
GRO^b^	457.6±150.9	486.6±171.1	509.4±203.9
MCP-3^b^	12.9±6.5	11.9±8.4	11.1±6.7
MDC^b^	872.0±259.4	977.2±243.4	1076.0±335.9
sIL-2Rα^b^	15.6±11.5	34.0±34.3	22.9±16.0
sCD40L^b^	10168.0±1793.0	10172.0±1844.0	10619.0±45.7
Eotaxin-2^c^	1011.0±639.8	1409.0±648.3	1186.0±1078.0
Eotaxin-3^c^	12.2±8.3	10.3±3.2	14.1±17.4
IL-23^c^	79.9±68.8	405.0±0.0	155.7±196.6

± SD. If *p*<0.01 by Kruskal-Wallis test, Mann-Whitney pairwise comparisons were performed. *p<0.05, **p<0.01, ***p<0.001 vs. control; §*p*<0.05, §§*p*<0.01, §§§*p*<0.001 vs. quiescent UC. ^a^ *n* = 34 controls, 28 quiescent UC, and 81 active UC. ^b^ *n* = 16 controls, 26 quiescent UC, and 31 active UC. ^c^ *n* = 34 controls, 28 quiescent UC, and 81 active UC. UC activity determined by histology. Values are in pg/ml. Mean

### Multiple cytokines/chemokines are increased in active UC tissues vs. control tissues

**Figure 2:**
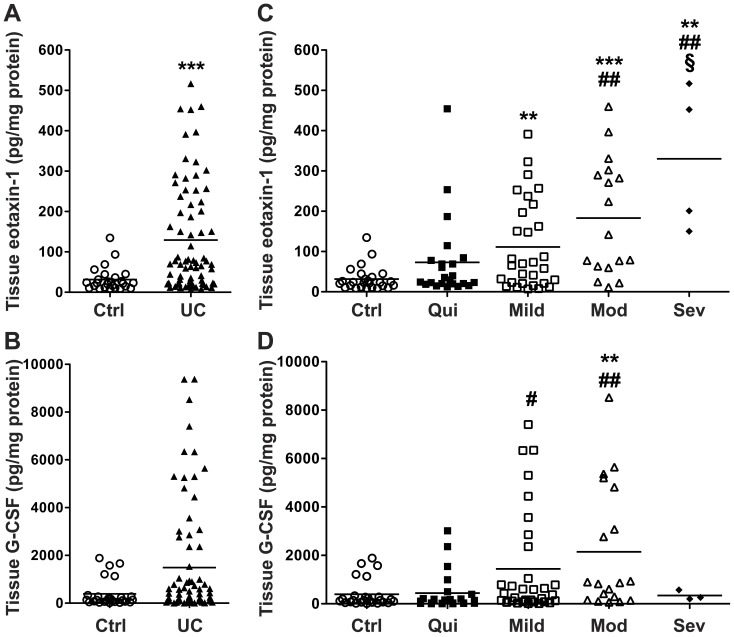
Colon tissue eotaxin-1 is significantly increased in UC. Snap frozen colonic biopsies were lysed and cytokine/chemokine levels were measured by Luminex technology, with each sample corrected for tissue lysate protein concentration, all as described in the Methods. (A) Eotaxin-1 and (B) G-CSF concentration in control and all UC patients. (C) Eotaxin-1 and (D) G-CSF concentration in UC tissues stratified by histologic disease severity, and in control subjects. *n* = 34 for control, and *n* = 86 for UC. **p<0.01; ***p<0.001 vs. control. ^#^ *p*<0.05; ^##^ *p*<0.01 vs. quiescent colitis. §*p*<0.05 vs. mild colitis.

In contrast to the serum, tissue cytokine/chemokine profiling revealed that 13 of 42 analytes (eotaxin-1, G-CSF, IP-10, IL-6, TNF-α, IL-17, MCP-1, MIP-1α, MIP-1β, IL-1α, IL-1β, IL-1RA, IL-8) were significantly increased in active UC vs. control tissues (Table 3). Of these, all but IL-1β were increased in tissues with active vs. quiescent colitis (Table 3). Eotaxin-1, but not G-CSF, was significantly increased when all UC patients were compared to control subjects (Figure 2A and 2B). When UC patients were grouped based on their tissue histopathology, eotaxin-1 was increased in mild, moderate, and severe disease and there was a progressive increase with worsening disease severity (Figure 2C). Tissue eotaxin-1 was significantly increased in moderate and severe colitis tissues vs. quiescent colitis, as well as in severe disease vs. mild disease (Figure 2C). G-CSF was only increased in moderate disease vs. control subjects as well as in mild and moderate colitis vs. quiescent colitis (Figure 2D).

**Table 3:** Tissue cytokines/chemokines profiles.

	Control	Quiescent UC	Active UC
Eotaxin-1^a^	31.6±29.3	73.1±21.3	155.1±19.8***§
G-CSF^a^	389.4±570.4	442.4±801.5	1787.0±2509.0**§§
IP-10^a^	93.1±46.1	182.1±215.8	902.2±1153.0***§§§
IL-6^a^	5.6±4.4	5.8±4.6	18.3±21.9**§§
TNF-α^a^	5.4±2.6	8.1±7.1	19.1±19.3***§§
IL-17^a^	10.0±6.3	13.1±9.5	45.4±42.6***§§§
MCP-1^a^	39.0±17.6	172.0±121.3***	609.2±785.7***§§§
MIP-1α^a^	16.7±7.6	33.3±44.5	70.8±69.3***§§
MIP-1β^a^	22.9±7.7	30.2±14.8	71.6±72.8***§§
IL-1α^a^	9.3±5.6	13.7±11.4	38.0±45.2***§
IL-1β^a^	7.5±3.1	13.1±9.7*	51.6±76.9***
IL-1RA^a^	324.7±374.3	429.2±335.9*	981.0±984.7***§§
IL-8^a^	24.9±25.8	37.4±29.6*	643.2±1248.0***§§§
GM-CSF^a^	5.7±2.5	8.3±4.9	13.6±13.2
IFN-γ^a^	6.2±6.6	21.0±12.2	32.2±28.8
IFN-α2^a^	43.1±15.9	54.4±32.0	62.3±52.3
IL-2^a^	24.0±9.7	35.4±20.2	38.6±41.2
IL-3^a^	158.5±195.1	521.1±831.6	182.1±194.6
IL-4^a^	50.2±48.4	221.9±826.2	95.8±135.4
IL-5^a^	3.0±1.7	7.0±13.0	9.4±12.8
IL-7^a^	20.0±10.6	22.8±13.0	40.3±46.1
IL-9^a^	7.7±4.0	9.9±4.1	13.9±9.7
IL-10^a^	970.8±1226.0	817.0±1143.0	1856.0±2462.0
IL-12p40^a^	38.5±15.3	67.7±40.7	60.8±53.1
IL-12p70^a^	17.2±8.1	23.6±13.8	33.6±37.5
IL-13^a^	17.9±8.6	14.7±12.5	15.2±13.8
IL-15^a^	88.4±59.9	105.6±74.8	142.0±127.2
TNF-β^a^	5.5±2.6	6.2±4.8	9.7±12.7
EGF^a^	12.0±14.6	15.6±20.8	23.8±27.0
VEGF^a^	114.7±62.7	126.9±110.2	205.1±171.8
FGF-2^b^	885.1±347.0	1009.0±450.9	877.3±521.9
TGF-α^b^	20.0±10.9	19.3±9.3	16.5±10.8
FLT-3L^b^	27.5±7.3	29.0±7.6	31.9±11.7
Fractalkine^b^	138.9±95.9	149.0±80.2	186.0±158.5
GRO^b^	143.9±147.8	283.6±325.4	246.6±164.1
MCP-3^b^	19.3±7.4	27.0±11.2	25.6±12.4
MDC^b^	107.1±64.6	128.6±79.5	189.1±149.1
sIL-2Rα^b^	72.7±41.5	99.6±59.9	184.4±178.1
sCD40L^b^	1859.0±638.5	1876.0±752.3	2215.0±694.5
Eotaxin-2^c^	670.4±460.0	998.5±1163.0	1295±1247.0
Eotaxin-3^c^	242.7±227.6	122.9±228.7	335.1±403.8
IL-23^c^	141.8±107.0	117.0±136.5	105.5±114.9

± SD. Statistical analysis performed as in Table 2. *p<0.05, **p<0.01, ***p<0.001 vs. control; §*p*<0.05, §§*p*<0.01, §§§*p*<0.001 vs. quiescent UC. ^a^ *n* = 34 controls, 26 quiescent UC, and 60 active UC. ^b^ *n* = 20 controls, 19 quiescent UC, and 23 active UC. ^c^ *n* = 34 controls, 26 quiescent UC, and 60 active UC. UC activity determined by histology. Values are in pg/mg protein. Mean

### Eotaxin-2 and eotaxin-3 are not altered in the serum or tissues of UC patients

**Figure 3:**
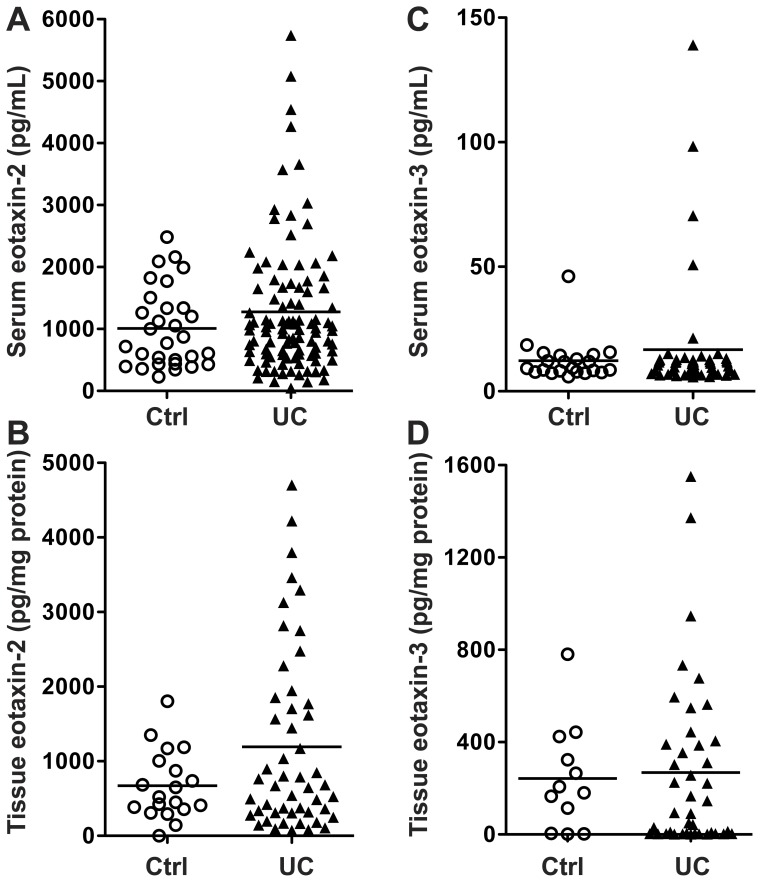
Eotaxin-2 and -3 levels in UC. Serum and tissue cytokine/chemokine levels are based on Luminex assay using a custom kit for eotaxin-2 and eotaxin-3. (A – B) Serum levels. (C – D) Tissue levels. For serum, *n* = 33 for control and *n* = 101 for UC. For tissue, *n* = 18 for control and *n* = 50 for UC. Note that many samples did not have reliable eotaxin-3 results.

Because of the consistent changes in eotaxin-1 in the serum and tissue samples from the UC patients, we then further assessed these samples for the closely related proteins eotaxin-2 and eotaxin-3, using Luminex technology. There was no significant increase in the serum (Table 2) or tissue (Table 3) levels of either protein in active UC vs. controls. When quiescent and active UC cases were combined (Figure 3A–D), it was notable that there were some cases, especially for eotaxin-2 (Figure 3A and 3B) that exhibited higher levels in the UC sera and tissues, but the overall differences were not statistically significant.

### Eotaxin-1 mRNA expression is increased in UC patients vs. control subjects and is also increased in areas of active disease vs. inactive disease in the same UC patient

**Figure 4:**
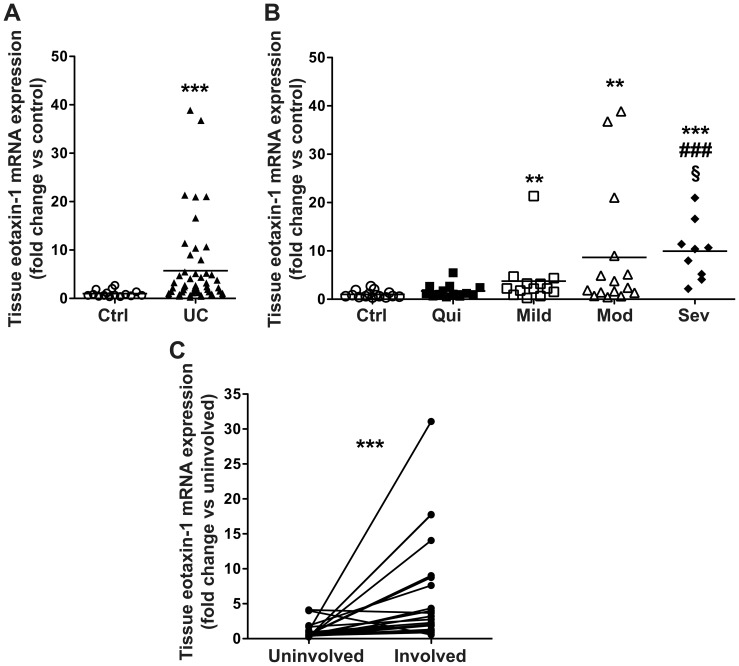
Eotaxin-1 mRNA expression is increased in UC tissues vs. control subjects and in involved vs. uninvolved tissues in UC patients with colitis limited to the left colon. Tissue samples were snap frozen, and subsequently RNA was extracted and mRNA expression assessed by real-time PCR. (A) Eotaxin-1 mRNA levels in control and all UC patients. (B) Eotaxin-1 mRNA levels stratified by UC histologic disease severity. *n* = 22 for controls, and *n* = 46 for UC patients. **p<0.01; ***p<0.001 vs. control. ^###^ *p*<0.001 vs. quiescent colitis. §*p*<0.05 vs. mild colitis. (C) Eotaxin-1 mRNA expression in paired uninvolved (right-sided) tissue and involved (left-sided) tissues. *n* = 21 paired samples from UC patients. ***p<0.001 vs. paired uninvolved tissue.

As eotaxin-1 was the only chemokine increased in both the serum and tissue of UC patients that continued to be significantly increased when patients were stratified by histologic disease severity, we also assessed the tissue eotaxin-1 mRNA expression. Eotaxin-1 mRNA levels were increased in UC patients vs. control subjects (Figure 4A). When grouped based on tissue histopathology, eotaxin-1 gene expression was increased in mild, moderate, and severe disease vs. control subjects. Tissues with severe UC activity exhibited increased tissue eotaxin-1 mRNA expression compared to either quiescent or mild disease (Figure 4B). Given the alterations in both tissue eotaxin-1 protein (Figure 2C) and mRNA expression (Figure 4B), we assessed eotaxin-1 mRNA expression in paired tissue samples from areas of active disease (involved) and normal colonic tissue (uninvolved) from the same UC patient in cases with left-sided colitis only. We found a 6.54±0.97–fold increase in eotaxin-1 mRNA expression in areas involved with left-sided colitis vs. uninvolved right colon (Figure 4C).

### Serum and tissue cytokines that are increased based on other indicators of disease severity

**Figure 5:**
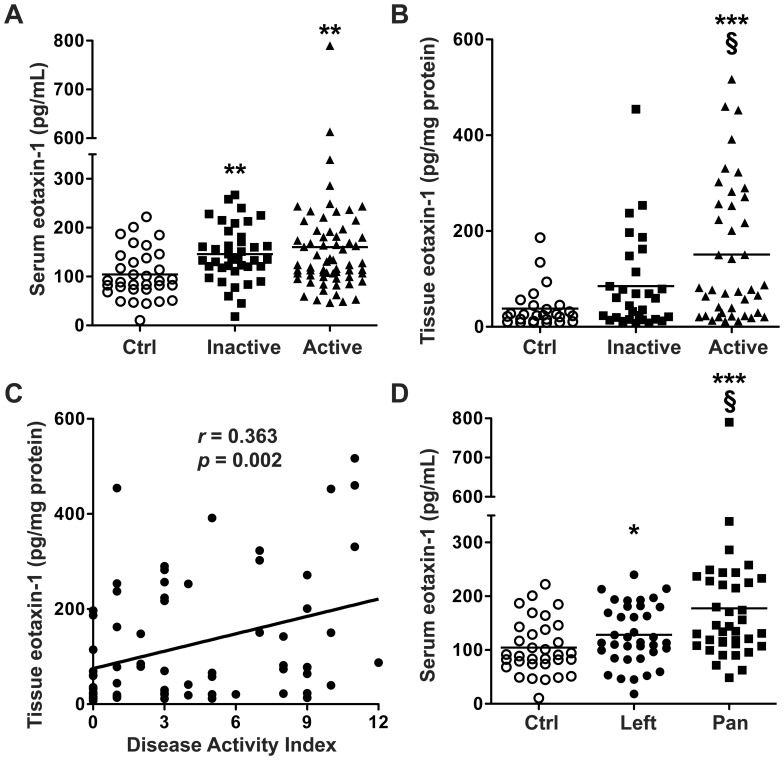
Serum and tissue cytokine/chemokine levels based on indices of disease severity. (A – B) Active disease is defined as a DAI >2, inactive disease as DAI 0 or 1. In A and B, for serum, *n* = 34 for control, and *n* = 103 for UC; for tissue, *n* = 34 for control, and *n* = 72 for UC. **p<0.01; ***p<0.001 vs. control. §*p*<0.05 vs. inactive disease. (C) Tissue eotaxin-1 is correlated with the DAI. In C, there are no data from control subjects undergoing colonoscopy for non-UC indications, as a DAI was not obtained in control subjects. (D) Serum eotaxin-1 is significantly increased in pancolitis patients vs. left sided-colitis patients. In D, *n* = 34 for control, *n* = 37 for left-sided colitis, and *n* = 38 for pancolitis. *p<*0.05*; ***p<0.001 vs. control. §*p*<0.05 vs. left-sided colitis. In A and D, the control samples are the same.

Given the increases in eotaxin-1 in the serum and tissues from UC patients with differing levels of histologic disease severity, we investigated the relationship between the Mayo Disease Activity Index (DAI), a validated clinical disease activity tool, and eotaxin-1 levels. When UC patients were classified by DAI into inactive (DAI 0 – 2) or active (DAI 3 – 12) disease, both serum and tissue eotaxin-1 levels were increased in active disease compared to the control subjects that were undergoing endoscopy for non-UC indications (Figure 5A and 5B). In tissues, eotaxin-1 was significantly increased in active vs. inactive disease (Figure 5B) and was positively correlated with the DAI in UC patients (Figure 5C). Of the 12 additional cytokines/chemokines increased in active UC tissues (Table 3), each was positively correlated with the DAI (*p* = 0.006 or less and a Spearman’s *r* value = 0.318 or greater for all), except G-CSF (data not shown). We then further assessed the effect of extent of active mucosal disease on serum eotaxin-1 levels by grouping patients into those with isolated left-sided colitis or pancolitis. Serum eotaxin-1 was significantly increased in both left-sided colitis and pancolitis vs. control patients, but there was a significant further increase in patients with pancolitis vs. left-sided colitis (Figure 5D).

### Tissue eosinophil counts are increased in UC patients and this correlates with tissue eotaxin-1 levels

**Figure 6:**
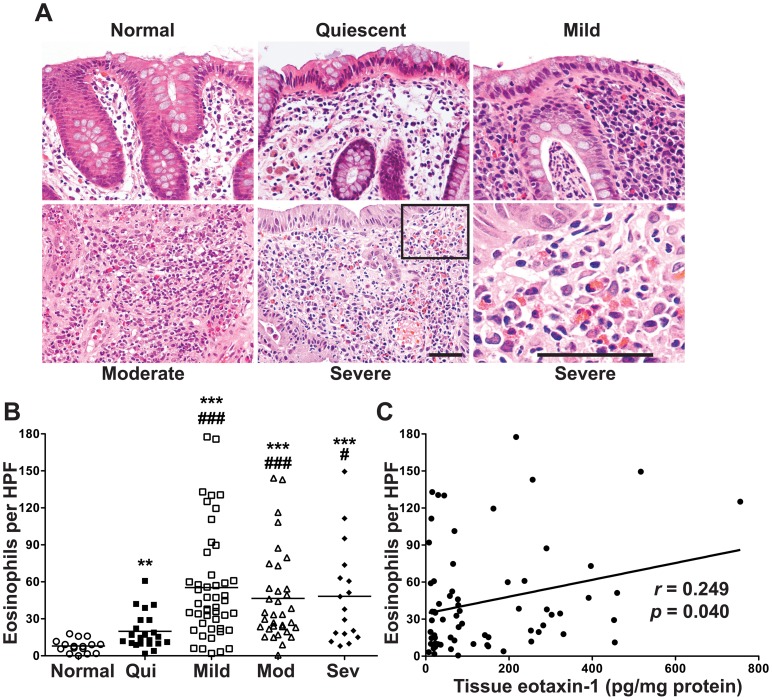
Increased tissue eosinophils in UC patients and relationship to tissue eotaxin-1 levels. As we did not have paraffin-embedded samples from the non-UC control subjects, we used a subset of 16 cases from our UC study patients who had completely normal histology in all of their colon biopsies at the time of the study colonoscopy as the normal group for this set of studies. (A) H&E sections from normal, quiescent, and active UC groups. (B) Number of eosinophils per HPF. **p<0.01; ***p<0.001 vs. normal. ^#^ *p*<0.05; ^###^ *p*<0.001 vs. quiescent colitis. *n* = 16 for normal, and *n* = 120 for UC. *C*: Correlation of tissue eotaxin-1 levels with eosinophil infiltration in active UC subjects with a β = 0.068. *n* = 68 paired samples.

We obtained the H&E slides for the UC patients to assess the tissue eosinophil counts in colonic mucosa. Representative photomicrographs are shown in Figure 6A. When grouped by histologic severity of disease, the tissue eosinophil counts were increased at all levels of disease vs. the tissues from UC patients with normal histology on all their study biopsies (Figure 6B). Patients with mild to severe colitis had increased tissue eosinophils vs. patients with quiescent colitis (Figure 6B). We also detected a positive correlation between tissue eotaxin-1 levels and tissue eosinophil counts (Figure 6C).

### Eotaxin-1 is increased in experimental models of murine colitis but not *H. pylori* gastritis

**Figure 7:**
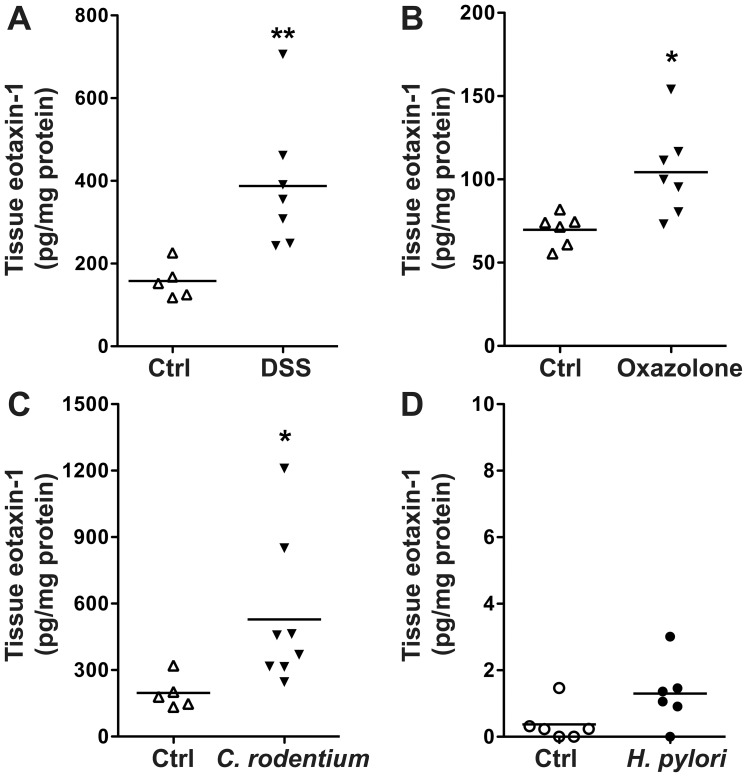
Colonic tissue eotaxin-1 levels are increased in mouse models of colitis but not in *H. pylori*-induced gastritis. Tissue samples were obtained at the time of sacrifice, placed in RIPA buffer, then lysed. Eotaxin-1 levels were assessed by Luminex assay. Each sample was corrected for tissue lysate protein concentration. Tissues were obtained as follows: (A) After 7 days of 4% DSS in the drinking water. (B) 3 days after intrarectal exposure to oxazolone. (C) 14 days after initiation of *C. rodentium* infection. (D) 4 months after inoculation with *H. pylori*. *p<0.05; **p<0.01 vs. control.

We have previously published that eotaxin-1 is increased in colonic tissues from mice exposed to DSS in an injury and recovery model of murine colitis [8]. When we assessed two murine models with similarities to human UC, namely the DSS model of acute injury and also the acute oxazolone model of colitis, we found increased eotaxin-1 in the colonic tissues (Figure 7A and 7B). Similarly, there was an increase in tissue eotaxin-1 in the *C. rodentium* infectious model of colitis (Figure 7C). However, when we accessed gastric tissues from mice infected with *H. pylori*, we did not detect a significant increase in eotaxin-1 levels (Figure 7D).

## Discussion

In this study, we utilized prospectively collected serum and colonic tissue from UC patients and control subjects to assess the utility of Luminex multiplex technology in defining altered cytokine and/or chemokine profiles in UC patients. We found that out of 42 analytes assessed in the serum, only 2 were significantly increased in patients with active UC vs. control subjects. When the UC patient serum was further categorized by histologic disease severity, only eotaxin-1 was increased at all levels of severity, whereas G-CSF was not increased. When colonic tissue samples were assessed using the same technique, there were multiple cytokines and chemokines that were significantly increased in active UC patients vs. control subjects, which continued to be increased when UC patients were stratified by histologic disease severity. However, only eotaxin-1 was increased at all levels of active disease in both serum and UC tissues. We further show that eotaxin-1 mRNA levels were increased in UC vs. control tissues, as well as when stratified by histologic disease severity, and there was an increase in the involved left colon vs. the paired uninvolved right colon from UC patients with left-sided colitis. Tissue eotaxin-1 was significantly correlated with the DAI. When categorized by the extent of mucosal disease activity, there was an increase in the serum eotaxin-1 from patients with either left-sided colitis or pancolitis vs. control patients, but this was significantly further increased in patients with pancolitis vs. left-sided colitis, further indicating the potential utility of eotaxin-1 measurement in patient stratification.

The utilization of Luminex technology to delineate the systemic and/or mucosal inflammatory response associated with colonic inflammation has been reported in only 6 papers in the literature. Four of these 6 are studies from our group in which the technique was utilized to investigate immune/inflammatory responses in experimental murine colitis [7], [8], [9], [10]. In humans, a recent study used Luminex assays to assess mucosal cytokines in microscopic colitis patients, where 10 analytes were examined in 10 control patients, 3 UC patients, and 25 microscopic colitis patients [25]; increases in IL-6 and IL-21 protein were detected in collagenous colitis, but no increases in any analytes were detected in UC tissues, due most likely to the small number of patients studied. Another recent report describes utilizing Luminex assays in the sera of 99 UC patients, but only 4 targets were examined, with no control patients used for comparison in this part of the study [26]. Of the 4 targets assessed in that study, only IL-17A was increased in severe disease vs. mild or moderate disease as determined by DAI [26]. Interestingly, in our analysis, serum IL-17 was significantly increased in active UC (determined by histology) compared to quiescent UC, but the level in active UC was not significantly increased compared to control subjects (Table 2).

Serum eotaxin has been shown to be increased in adult IBD patients compared to control subjects [27], [28]. In each of these two studies there were 35 UC patients, less than half with active disease, compared to the 137 UC cases in our report, where 96 had active disease by histologic criteria. Additionally, these studies focused only on eotaxin-1 and/or eotaxin-2 using single analyte solid phase sandwich enzyme-linked immunosorbent assays (ELISAs), rather than Luminex assay, where we were able to determine the relative importance of eotaxin-1 vs. 41 other cytokines/chemokines. Neither of these earlier studies examined tissue eosinophils, whereas we demonstrated increased eosinophils in UC vs. normal tissues, increased levels in each of the active stages of colitis vs. quiescent colitis, and a correlation of eosinophil counts with eotaxin-1 levels. Chen *et al*. [27] also measured eotaxin-2 in the serum and tissues, and found no increase in UC compared to controls. This finding is similar to our data, although we took the approach of studying eotaxin-2 and eotaxin-3 in both serum and tissues and found no increases in the UC cases. These studies examined 31 [27] and 37 [27], [28] Crohn’s disease patients, and found increased levels of serum eotaxin-1 compared to controls, but these levels were not different from UC cases. Thus, to further assess the potential involvement of eotaxin-1 in Crohn’s disease, a larger cohort, similar to the size of our current UC study, and comparison to tissue eosinophils, as we have performed, is needed.

More recently, eotaxin-1 has been shown to be increased in the sputum and intestinal biopsies of pediatric patients with asthma and eosinophilic gastrointestinal diseases [29]. Additionally, tissue eotaxin-1 mRNA levels have been shown to be increased in a study of 13 pediatric UC cases [30] compared to controls, which was similar to our findings that eotaxin-1 mRNA expression was increased in 46 adult UC cases studied that were compared to 22 controls. We extended our observations to show that in paired involved and uninvolved tissues eotaxin-1 mRNA levels were increased in active disease. None of the previous studies compared uninvolved and involved tissues from the same patients. The study by Ahrens *et al*. [30] indicated that eotaxin-1 mRNA levels by gene-chip analysis correlated with tissue eosinophil levels when assessed in 8 pediatric UC patients. That same report also showed by immunofluorescence staining that eotaxin-1 was present in both the colonic macrophages and epithelial cells in the UC patients [30]. We also demonstrated increased eosinophil counts in UC tissues in our study, where we compared 16 patients with normal histology to 120 UC cases. Additionally, in 69 cases with active UC, we demonstrated a correlation of tissue eotaxin-1 protein levels and eosinophil counts.

The presence of eosinophilic inflammation has been recognized in patients with other chronic inflammatory processes leading to the identification of distinct subgroups that may benefit from a more targeted therapeutic approach. For example, persistent eosinophilia (both peripheral and sputum) has been recognized as a hallmark of a subset of adult onset, severe asthma patients who are resistant to steroid therapy [31]. Recurrent peripheral eosinophilia has also been associated with increased UC severity and a predisposition for primary sclerosing cholangitis [32]. We were able to assess the peripheral blood eosinophil counts (as a percentage of total blood cell count) obtained around the time of the study colonoscopy in 6 control subjects and 66 UC patients in our case series. There was no significant difference between serum eosinophils in control (2.3±0.5%) and UC patients (2.8±0.3%) in the current cohort (data not shown).

A potential confounding factor in this study is that 92.7% of the UC patients were on at least one therapy (Table 1). When stratified based on specific medication exposures, patients treated with corticosteroids had significantly higher tissue, but not serum, eotaxin-1 levels, suggesting that this treatment may have contributed to a reduction in circulating levels (Table S1 and S2). In contrast, those on anti-TNF-α therapies had somewhat decreased tissue eotaxin-1 levels, though the later did not reach statistical significance; also no effect was seen with 5-aminosalicylates or immunomodulators (Table S1 and S2). Additionally, 8 UC patients were felt to be inappropriate candidates for the biopsy protocol at the time of colonoscopy due to endoscopically severe disease; therefore no study samples were obtained, which may have been a bias that could have reduced our ability to detect differences in cytokines/chemokines in UC vs. controls. Another limitation was that patients were studied at a single time point and that longitudinal follow-up was not part of the study design.

Consistent with our findings in UC colitis tissues, we also found that eotaxin-1 was increased in the murine DSS epithelial injury model [8]. It has been shown that eotaxin-1 deficient mice were protected from clinical colitis in the DSS model, with decreased body weight loss, nearly normal colon length, and a decrease in tissue eosinophils [33], suggesting that eotaxin-1 may contribute to the causation of colitis in this model system. We also found significant increases in eotaxin-1 in tissues from the oxazolone and *C. rodentium* colitis models. However, there was no increase in *H. pylori*-induced gastritis tissues, thus, the increased eotaxin-1 levels do not appear to be a non-specific component of the mucosal inflammatory response. We have previously shown that L-arginine can improve clinical and biochemical parameters in a DSS-induced colitis model of injury and repair, and that eotaxin-1 was one of the proinflammatory mediators that was reduced with the L-arginine treatment [8].

In summary, the present study provides evidence that Luminex technology is sufficiently robust to identify multiple proinflammatory cytokines/chemokines that are increased in the serum and tissue of active UC patients compared to control subjects. Interestingly, eotaxin-1 was the only analyte significantly increased in both serum and tissues in active UC; it was also increased in murine colitis models but not *H. pylori*-induced gastritis tissues. Our finding correlating tissue eotaxin-1 protein with eosinophil counts in active UC defines a subgroup of patients in which therapy directed against eotaxin-1 may be most effective. Our data suggest that Luminex profiling may be useful in selecting patients for new therapies, such as an anti-eotaxin-1 monoclonal antibody, for which a phase II clinical trial is being initiated (NCT01671956, ClinicalTrials.gov), and for following responses to this and other treatments. We suggest that in the specific case of anti-eotaxin-1 antibody, it will be interesting to learn if eotaxin-1 levels are useful in predicting clinical response, and this strategy could lead to more effective clinical application of new therapies.

## Supporting Information

Table S1 **Serum eotaxin-1 levels in UC patients based on medication use.** Serum samples were obtained and assessed by Luminex as in Figure 1. UC patients were categorized by medication use at the time of serum collection.
(DOC)

Table S2 **Tissue eotaxin-1 levels in UC patients based on medication use. Tissue samples were obtained and assessed by Luminex as in** **Figure 2** **.** UC patients were categorized by medication use at the time of tissue collection.
(DOC)
