# Supplementary material for: High-Throughput Multi-Analyte Luminex Profiling Implicates Eotaxin-1 in Ulcerative Colitis
Source: PLoS One. 2013 Dec 18;8(12):e82300. doi: 10.1371/journal.pone.0082300 (PMC3867379; doi:10.1371/journal.pone.0082300)
Supplement: Table S1 — Serum eotaxin-1 levels in UC patients based on medication use. Serum samples were obtained and assessed by Luminex as in Figure 1. UC patients were categorized by medication use at the time of serum collection. (DOC) [file pone.0082300.s001.doc]

**Table S1. Serum eotaxin-1 levels in UC patients based on medication use.**

| No 5-ASA (*n* = 20) | 5-ASA (*n* = 85) | No Steroids (*n* = 88) | Steroids (*n* = 17) | No Immunomodulator (*n* = 67) | Immunomodulator (*n* = 38) | No Anti-TNF-α (*n* = 88) | Anti-TNF-α (*n* = 17) |
| --- | --- | --- | --- | --- | --- | --- | --- |
| 160.6 ± 62.0 | 153.4 ± 104.6 | 154.7 ± 103.9 | 141.0 ± 57.9 | 159.8 ± 116.1 | 146.9 ± 55.2 | 153.8 ± 103.0 | 161.7 ± 70.3 |

Values are presented as pg/ml. Mean ± SD. Mann-Whitney pairwise comparisons were performed.
