# Supplementary material for: High-Throughput Multi-Analyte Luminex Profiling Implicates Eotaxin-1 in Ulcerative Colitis
Source: PLoS One. 2013 Dec 18;8(12):e82300. doi: 10.1371/journal.pone.0082300 (PMC3867379; doi:10.1371/journal.pone.0082300)
Supplement: Table S2 — Tissue eotaxin-1 levels in UC patients based on medication use. Tissue samples were obtained and assessed by Luminex as in Figure 2 . UC patients were categorized by medication use at the time of tissue collection. (DOC) [file pone.0082300.s002.doc]

**Table S2. Tissue eotaxin-1 levels in UC patients based on medication use.**

| No 5-ASA (*n* = 12) | 5-ASA (*n* = 60) | No Steroids (*n* = 56) | Steroids (*n* = 16) | No Immunomodulator (*n* = 52) | Immunomodulator (*n* = 20) | No Anti-TNF-α (*n* = 60) | Anti-TNF-α (*n* = 12) |
| --- | --- | --- | --- | --- | --- | --- | --- |
| 155.0 ± 132.7 | 125.1 ± 135.5 | 109.5 ± 127.7 | 200.4 ± 137.3^a^ | 126.5 ± 138.7 | 139.6 ± 126.3 | 141.9 ± 142.7 | 73.7 ± 61.3 |

Values are presented as pg/mg protein. Mean ± SD. Mann-Whitney pairwise comparisons were performed. ^a^*p* < 0.01 vs no steroids.
